# Differential Expression Spectrum of circRNA in Plasma Exosomes in Dilated Cardiomyopathy With Heart Failure

**DOI:** 10.1111/jcmm.70258

**Published:** 2024-12-24

**Authors:** Shuai Xu, Ge Zhang, Xin Tan, Yiyao Zeng, Huimin Fan, Jiamin Gao, Zhen Qin, Fengyi Yu, Bin Ma, Ting Zhang, Hezi Jiang, Xian Li, Xiangyu Wang, Jili Fan, Xiaohong Bo, Yafeng Zhou, Junnan Tang

**Affiliations:** ^1^ Department of Cardiology The First Affiliated Hospital of Zhengzhou University Zhengzhou China; ^2^ Henan Province Key Laboratory of Cardiac Injury and Repair Zhengzhou China; ^3^ Henan Province Clinical Research Center for Cardiovascular Diseases Zhengzhou China; ^4^ Department of Cardiology, The Fourth Affiliated Hospital of Soochow University, Suzhou Dushu Lake Hospital Medical Center of Soochow University Suzhou China; ^5^ Institute for Hypertension Soochow University Suzhou China; ^6^ Center of Translational Medicine and Clinical Laboratory The Fourth Affiliated Hospital to Soochow University Suzhou China; ^7^ Luoyang Central Hospital Affiliated of Zhengzhou University Luoyang China; ^8^ Department of Cardiology, the Second People's Hospital of Hefei Hefei Hospital Affiliated to Ahhui Medical University Hefei China; ^9^ Department of Cardiovascular Disease Taihe County People's Hospital Fuyang China

**Keywords:** circRNA, dilated cardiomyopathy, heart failure, plasma exosomal

## Abstract

Dilated cardiomyopathy (DCM), a form of non‐ischaemic myocardial disease, is characterised by structural and functional cardiac abnormalities. As defined by the World Health Organisation, DCM constitutes a significant cardiac pathology, leading to increased morbidity and mortality due to complications such as heart failure and arrhythmias. The diagnostic process for DCM predominantly employs echocardiography and MRI, with biomarkers like NT‐pro BNP and troponin providing supportive, yet non‐specific, evidence. Exosomes, small extracellular vesicles, play a critical role in intercellular communications by transferring biomolecules including lipids, proteins, messenger RNA (mRNA) and non‐coding RNA (ncRNA) to target cells, thereby influencing key cellular processes such as proliferation, differentiation, apoptosis, angiogenesis and immune modulation. Within the ncRNA category, circular RNAs (circRNAs) are notable for their cellular specificity and evolutionary conservation and are often implicated in the regulatory mechanisms underlying DCM and heart failure. This investigation employed next‐generation sequencing technology to analyse plasma exosomal circRNA profiles in DCM patients with chronic heart failure (CHF), compared to healthy controls. The analysis revealed distinct circRNA expression patterns, identifying 49 uniquely expressed circRNAs in the DCM cohort with CHF. These circRNAs were associated with several critical biological pathways, including the sequestration of extracellular ligands from receptors, N‐acetyltransferase activity, histone acetyltransferase activity and endocytic vesicle membrane composition. The findings of this study provide valuable insights into the pathophysiological mechanisms of DCM and offer evidence for improving clinical diagnostic methodologies.

## 1 Introduction

Dilated cardiomyopathy (DCM) is a non‐ischaemic myocardial disease characterised by cardiac muscle's structural and functional abnormalities. Its clinical symptoms are ventricular dilation and systolic dysfunction, typically in the absence of coronary artery disease, hypertension, valvular disease or congenital heart disease [1]. The World Health Organisation categorises DCM as a severe cardiac illness due to its high morbidity and mortality, primarily through heart failure and arrhythmias [2]. Epidemiological studies estimate that the prevalence of DCM is about 1 in 250 individuals, surpassing the incidence rates of hypertrophic cardiomyopathy (HCM) and arrhythmogenic right ventricular cardiomyopathy (ARVC) [3]. With an annual incidence of approximately 5–8 per 100,000, DCM is likely underdiagnosed and often occurs as a final common phenotype in diverse cardiac diseases. Notably, DCM accounts for up to 40% of heart failure patients with reduced ejection fraction, requiring hospitalisation or evaluation for heart transplantation [4].

The aetiology of DCM is multifaceted, involving genetic mutations, infection, inflammation, autoimmune disorders, exposure to toxins and endocrine or neuromuscular dysfunctions [5, 6]. DCM is highly hereditary, with genetic factors accounting for 30%–50% of all cases [7]. Notable genetic associations have been identified, including truncation variants of the Titin (TTNtv) gene, which impact the giant sarcomeric protein titin, and is the most prevalent genetic cause of DCM [8]. Additionally, mutations in the Lamin A/C (LMNA) gene are associated with an increased risk of sudden cardiac death [9], mutations in the myosin heavy chain (MYH7) gene are linked to early onset and pronounced phenotypic expression [10], and truncation mutations in the Filamin C (FLNC) gene lead to haploinsufficiency, associated with both DCM and arrhythmias [11]. Mutations in other genes such as troponin‐T (TNNT2), troponin‐C1 (TNNC1), phospholamban (PLN) and desmoplakin (DSP) have also been implicated in the development of DCM [12, 13]. However, the specific mechanisms by which these genetic variations contribute to the pathophysiology of DCM are not yet fully elucidated. With the advancement of next‐generation sequencing technologies, an increasing number of genes related to DCM are being discovered, offering significant implications for early clinical diagnosis.

Exosomes are recognised as a category of extracellular vesicles (EVs) derived from multiple cell types and present in various biological fluids, including serum, plasma, urine, saliva, ascites, cerebrospinal fluid and amniotic fluid [14]. These vesicles, approximately 30–150 nm in diameter, play a pivotal role in cell‐mediated communications [14, 15]. While previously regarded as mere evolutionary byproducts, ncRNAs are now acknowledged to play crucial roles in various biological processes through epigenetic, translational and post‐transcriptional mechanisms. CircRNAs, with their unique covalently closed loop structures, exhibit resistance to RNase R degradation, conferring them better stability than linear RNAs [16]. Recent studies have unveiled a multitude of functions of circRNAs, among which their role as competing endogenous RNAs (ceRNAs) has become a focal point of research [17]. For instance, the study by Zeng et al. highlighted the regulatory effect of CircMAP3K5 on cardiovascular intimal hyperplasia–related diseases [18]. Due to the higher abundance, quality and stability, exosome‐derived ncRNAs serve as a superior source for biomarker research. In a review written by Kamal and Shahidan, which analysed 32 studies involving exosomal and non‐exosomal miRNAs, miRNAs from exosomes have been identified as a better source of biomarkers [19]. Furthermore, approximately 75% of articles studying on ncRNAs recommend the utilisation of exosome‐derived ncRNAs over non‐exosomal ncRNAs [20]. Therefore, exosome‐derived ncRNAs are regarded to significantly improve disease screening and monitoring, offering promising prospects in disease diagnosis and therapy. Nevertheless, current research on the role of exosome‐derived circRNAs in early diagnosis of DCM remains relatively limited [21].

In this study, we explored the circRNA expression spectrum in plasma exosomes from DCM patients with chronic heart failure (CHF). By employing next‐generation sequencing and bioinformatics, we identified differentially expressed exosomal circRNAs and analysed their potential roles in DCM pathophysiology. Our findings offer new perspectives on DCM diagnosis and treatment and provide the foundation for further circRNA research in plasma exosomes.

## 2 Materials and Methods

### 2.1 Participants

This study, approved by the Institutional Review Board of the First Affiliated Hospital of Zhengzhou University and the Fourth Affiliated Hospital of Soochow University (Approval No. 2020‐KY‐142), recruited patients with DCM combined with CHF from the First Affiliated Hospital of Zhengzhou University and the Fourth Affiliated Hospital of Soochow University. The cohort included five healthy volunteers and three DCM patients. DCM diagnosis adhered to the criteria established by the European Society of Cardiology [22]. Cardiac functional status was assessed using the New York Heart Association (NYHA) functional classification system [23]. The general clinical characteristics of all participants are shown in Table S1.

Inclusion criteria for DCM and HF patients were (1) confirmed DCM diagnosis based on the European Society of Cardiology's 2016 guidelines [22], (2) symptoms of heart failure, aligning with the consensus guidelines for the CHF diagnosis, (3) a history of congestive heart failure (CHF) for over 6 months and (4) provision of signed informed consent. Exclusion criteria were (1) initial presentation with acute heart failure, (2) loss of consciousness, (3) severe infectious diseases or malignant tumours, (4) hospitalisation for less than 2 days and (5) incomplete clinical data.

### 2.2 Plasma Sample Collection

Patients diagnosed as DCM combined with CHF, along with healthy volunteers, were recruited for the study. Venous blood samples were collected in Na‐EDTA tubes to inhibit coagulation. Blood samples were centrifuged at 3000 *g* for 15 min at a 4°C temperature. Subsequently, the top 3 mL of plasma from each sample was rapidly frozen at −80°C to preserve the stability and integrity of plasma biomolecules.

### 2.3 Exosome Isolation

Exosomes were extracted from the plasma using the ExoQuick reagent (EXOQ5A1; System Biosciences, USA), adhering strictly to the manufacturer's protocol. Initially, 250 μL of plasma was mixed with 36 μL of ExoQuick Exosome Precipitation Solution and incubated at 4°C for 30 min. Centrifugation at 1500 *g* for 30 min followed. After discarding the supernatant, an additional centrifugation step at 1500 *g* for 5 min was performed. The resultant exosome pellet was resuspended in 100 μL of sterile phosphate‐buffered saline (PBS) and stored at −80°C for subsequent analysis.

### 2.4 Transmission Electron Microscopy

The morphology of the isolated exosomes was examined via transmission electron microscopy (TEM). Initially, exosomes were diluted in PBS and placed on a carbon‐coated copper grid. The grid remained undisturbed for 10 min, allowing exosome adherence. Excess fluid was then absorbed using filter paper. Samples were fixed in 3% glutaraldehyde for 5 min, followed by repeated rinsing with deionised water (10 times for 2 min each). Staining with 4% uranyl acetate was conducted for 10 min, followed by a fixation in 1% methylcellulose for 5 min. After air‐drying at room temperature for 30 min, the samples were prepared for TEM analysis.

### 2.5 Flow Nanoanalyzer

The concentration and diameter distribution of extracted exosomes were determined by Flow NanoAnalyzer (FL Sciences).

### 2.6 Western Blot

Proteins were isolated from exosomes using ice‐cold RIPA lysis buffer (Solarbio, China). For protein electrophoresis, uniform protein aliquots (20 μg) were subjected to a 12.5% SDS‐PAGE gel. Post‐electrophoresis, proteins were transferred to 0.45 μm polyvinylidene fluoride membranes (Millipore, USA). Membranes were blocked with 5% non‐fat milk at room temperature for 2 h and subsequently incubated overnight at 4°C with primary antibodies: CD81 (#ab109201, Abcam, USA), CD63 (#ab134045, Abcam, USA) and Calnexin (#ab92573, Abcam, USA). This was followed by a 2‐h incubation at room temperature with horseradish peroxidase‐conjugated anti‐rabbit secondary antibodies (#ZB‐2301, Zhongshan Golden Bridge Biotechnology, China). Protein bands were visualised and quantified using an Amersham Imager 600 (GE Healthcare Life Sciences, USA) in conjunction with ImageJ software.

### 2.7 CeRNA Micoarray Method

Total RNA was extracted from serum EVs using the Plasma/Serum/Exosomal RNA Purification Kit (Norgen, N‐51000) according to the manufacturer's instructions. RNA concentration was determined using a NanoDrop ND‐2000 (Thermo Fisher Scientific), and RNA integrity was assessed with an Agilent Bioanalyzer 2100 (Agilent Technologies, Santa Clara, CA, USA). Following RNA extraction with the RNeasy Mini Kit (QIAGEN, 74106), 50 ng of total RNA was combined with a spike‐in (Agilent 5188‐5282) for reverse transcription using the Low Input Quick‐Amp Labeling Kit, one‐colour (Agilent, 5190‐2305). Subsequently, cDNA was transcribed to cRNA using T7 RNA polymerase and labelled with cyanine‐3‐CTP. Labelled cRNAs were purified using the QIAGEN RNeasy Kit (Qiagen, 74,106). Biotinylated cRNA targets (1.65 μg) were prepared with a 10X blocking agent and 25× fragmentation buffer (Gene Expression Hybridization Kit, Agilent 5188‐5242), incubated at 60°C for 30 min and then hybridised with the LC Human ceRNA Array V1.0 (4*180K, Design ID: 085202) at 65°C for 17 h. Post‐hybridisation, the microarray was scanned using an Agilent Scanner G5761A (Agilent Technologies, Santa Clara, CA, USA). Data extraction was performed with Feature Extraction software 12.0.3.1 (Agilent Technologies, Santa Clara, CA, USA), and raw data were normalised using the quantile algorithm in GeneSpring software (version 14.8, Agilent Technologies). Probes detected in at least 80% of one group were considered for subsequent analysis [24]. Differentially expressed (DE) lncRNAs and mRNAs were identified based on a *p*‐value ≤ 0.05 and |log_2_FoldChange| ≥ 1. Heatmaps were generated using the R package ‘heatmap’. The microarray procedure and data analysis were conducted by LC‐Bio Technologies (Hangzhou, China). This study selected circRNA as the main research object.

### 2.8 CeRNA Network Analysis Method

For predictive analysis, we utilised TargetScan (version 5.0) and miRanda (version 3.3a), setting the minimum TargetScan score at ≥ 50 and the miRanda energy threshold at < −10. TargetScan and miRanda are tools used for miRNA‐target prediction. TargetScan identifies biological targets of miRNAs by searching for conserved sites that match the miRNA seed region, while miRanda aligns miRNA sequences to potential targets based on complementarity and duplex free energy. We considered the intersection of predictions from both tools for further analysis. Our analysis involved using significantly differentially expressed mRNAs, miRNAs, lncRNAs and circRNAs to predict target relationships between miRNA and mRNA, lncRNA, and circRNA, respectively. Subsequently, we constructed ceRNA relationship pairs based on shared miRNAs. Using Cytoscape software, we visualised these ceRNA pairs in a network diagram, elucidating the regulatory relationships and providing a comprehensive overview of the interactions within our study's scope. This integrated approach facilitated a detailed understanding of the molecular mechanisms under investigation. As in previous studies, Gene Ontology (GO) Term Enrichment Analysis (http://www.geneontology.org/) and Kyoto Encyclopedia of Genes and Genomes (KEGG) Pathway Analysis (http://www.genome.jp/kegg/) were employed to predict the function of these target genes [25]. GO enrichment analysis categorises genes into hierarchical categories such as biological processes, cellular components and molecular functions, allowing researchers to understand gene functions and interactions in biological contexts. Metascape (http://metas cape.org) integrates data from multiple databases to perform pathway enrichment analysis, functional annotation and clustering of enriched terms, providing a comprehensive view of biological processes and molecular functions [26]. Protein–protein interaction (PPI) network analysis identifies interactions between proteins, helping us to visualise how proteins interact and their involvement in various biological processes. This is crucial for understanding protein functions and regulatory mechanisms. The combined use of these tools provides a multifaceted analysis of biological data, revealing complex regulatory mechanisms and interactions. This holistic approach enhances the understanding of disease pathogenesis by integrating miRNA‐target interactions, gene functional annotations, pathway analysis and protein interaction networks.

### 2.9 RNA Extraction and RT‐PCR Analysis for circRNAs

To validate the expression levels of 10 key circRNAs (hsa‐circ‐0047519, hsa‐circ‐0063766, hsa‐circ‐76,771, hsa‐circ‐0054400, hsa‐circ‐0019699, hsa‐circ‐0074153, hsa‐circ‐0075837, hsa‐circ‐0056066, hsa‐circ‐0026806, hsa‐circ‐0080908), quantitative real‐time PCR (RT‐PCR) was performed in an external cohort. Actin served as the internal reference gene. RNA (up to 5 μg) extracted with QIAGEN (miRNeasy Serum/Plasma Kit (50) No./ID: 217184) according to the manufacture's protocols was reverse‐transcripted to cDNA using the Two‐Step PrimeScript RT‐PCR kit (TaKaRa, 6210A). The cDNA was diluted to an appropriate concentration with nuclease‐free water for use. Amplification of cDNA was carried out using SYBR Green qPCR mix (Bimake, B21202) and detected with the LightCycler 480 II Real‐Time PCR System (Roche). Data were normalised to ACTIN and expressed as the fold change in the circRNA expression of DCM patients' samples relative to the healthy people.

### 2.10 Protein–Protein Interaction Network Construction and Hub Genes Identification

The PPI network was analysed using the Search Tool for the Retrieval of Interacting Genes (STRING) database [27]. PPI pairs with a combined score greater than 0.4 (medium confidence) were selected for network construction. The network was visualised using Cytoscape software (http://www.cytoscape.org/). CytoHubba, a plugin in Cytoscape, was employed to calculate the degree of each node [28]. Nodes with higher degrees of connectivity were indicative of highly interactive proteins or genes within the network. In our analysis, nodes with a degree greater than 5 were identified as hub genes.

### 2.11 Establishment of the miRNA–mRNA Regulatory Network

The circbank database (https://www.mirnet.ca) and the circinteractome database (https://circinteractome.nia.nih.gov/mirna_target_sites.html) were utilised for the visualisation and analysis of circRNA‐centric networks [29, 30]. These platforms facilitate the integration of existing knowledge with user data, aiding in the elucidation of circRNA functions. We constructed the circRNA–miRNA–mRNA network for DCM with CHF using Cytoscape software [31].

### 2.12 Dimensional Reduction Analysis

Dimensional reduction of internal transcriptomic data, focusing on circRNAs, was performed using the uniform manifold approximation and projection (UMAP), utilising the umap‐learn package (https://umap‐learn.readthedocs.io/en/latest/). To evaluate the discriminative ability of the identified hub circRNAs, principal coordinate analysis (PCoA) and analysis of similarities (ANOSIM) were conducted [32]. The Bray–Curtis dissimilarity matrix was computed using beta_diversity.py, and the Bray–Curtis diversity indices were calculated employing the R package Vegan, specifically using the vegdist function.

### 2.13 Calculation of the Global Difference Between a Pair of Expression Profiles

We applied two different methods to calculate the global difference between a pair of expression profiles: the Euclidean distance [33],

$$\text{RMSD}=\sqrt{\sum_{i=1}^{n}{\left(\log2_{2}x_{i}-\log2_{2}y_{i}\right)}^{2}/n}$$

where *x*_*i*_ and *y*_*i*_ are the expression of hub circRNA *i* over two expression profiles (DCM‐Exo and Health‐Exo) with *p* and *q* samples (*x* ^1^, *x* ^2^, …, *x* ^p^), (*y* ^1^, *y* ^2^, …, *y* ^q^).

### 2.14 Statistical Analysis

All statistical tests employed in this study were two‐sided. A *p*‐value of < 0.05 and a false discovery rate (FDR) of < 0.05 were considered indicative of statistical significance. Descriptive statistics for continuous variables adhering to a normal distribution were expressed as mean ± standard deviation [34]. For comparisons involving continuous variables, the Wilcoxon rank‐sum test or Student's *t*‐test was applied as appropriate. Categorical variables were analysed using the chi‐squared test or Fisher's exact test. All processes of data handling, statistical analyses and graph plotting were executed using R software (version 4.1.3).

## 3 Results

### 3.1 Identification of Isolated Plasma Exosomes

**FIGURE 1:**
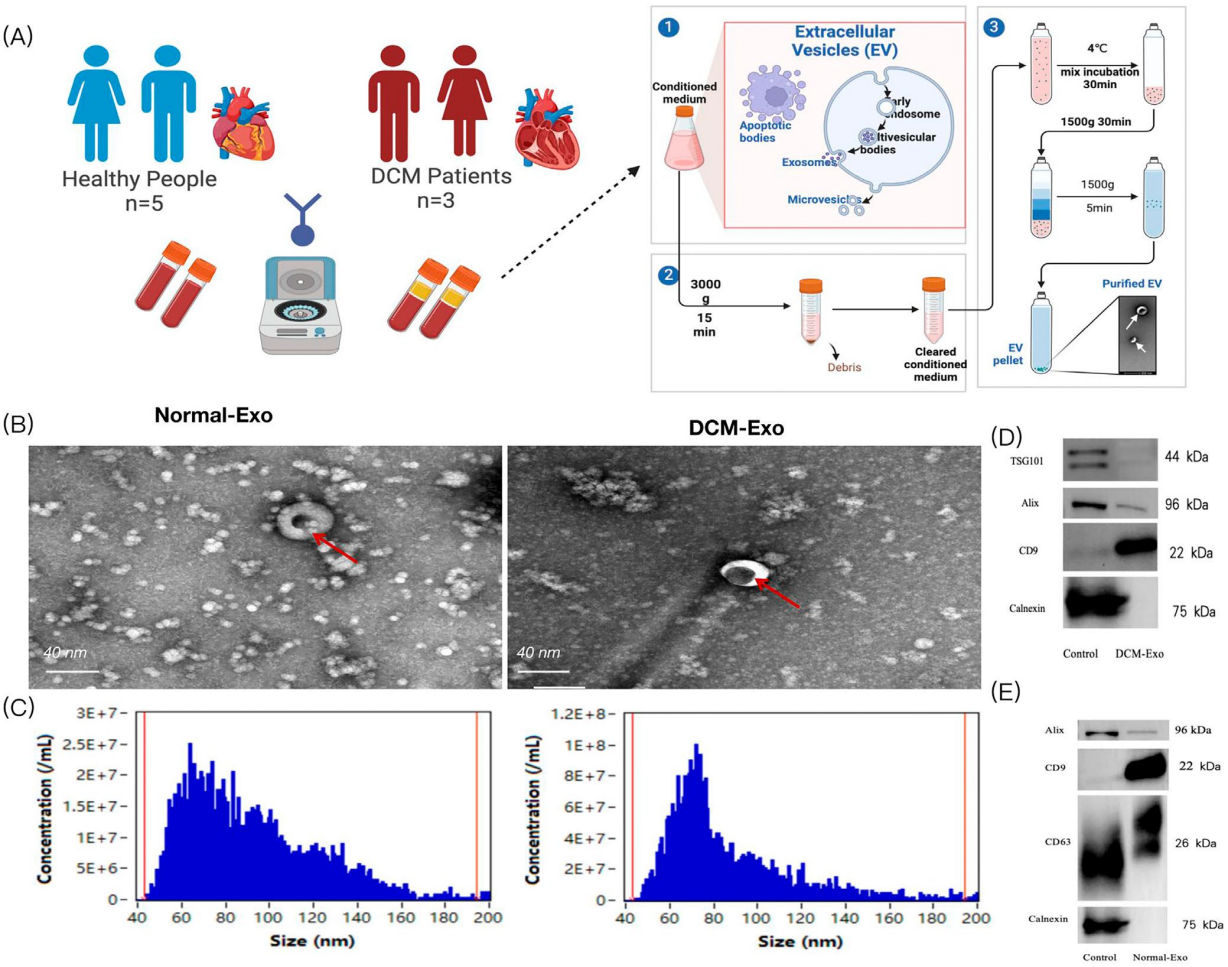
Characterisation and internalisation of DCM‐Exo and Nor‐Exo. (A) Complete workflow for high‐throughput sequencing and analysis of plasma exosome samples of DCM with CHF. Healthy group (*n* = 5) and DCM group (*n* = 3). (B) Electron microscopic images of isolated exosomes. Scale bar, 40 nm. Exosomes are indicated by red arrows. (C) Exosome size distribution and concentration. (D, E) Western blotting revealed CD9, CD63 proteins and EV negative marker protein Calnexin in exosome and cell control samples.

The study's methodology is depicted in an overarching flowchart (Figure 1A). Plasma‐derived exosomes were characterised based on diameter, morphology and surface protein expression, specifically CD63 and CD9. TEM images (Figure 1B) revealed that the isolated plasma exosomes exhibited typical cup‐shaped structures. Nanoparticle analysis using high‐sensitivity flow cytometry indicated median diameters of 82 nm for DCM‐exosomes (DCM‐Exo) and 77 nm for normal‐exosomes (Nor‐Exo), as shown in Figure 1C. Furthermore, the Western blot analysis confirmed a positive expression of CD63 and CD9 proteins and a negative expression of Calnexin in these exosomes (Figure 1D,E). This comprehensive characterisation enabled the accurate identification of plasma exosomes for subsequent high‐throughput sequencing.

### 3.2 Exosomal circRNA Profile of DCM With CHF Patients by ceRNA Sequencing

**FIGURE 2:**
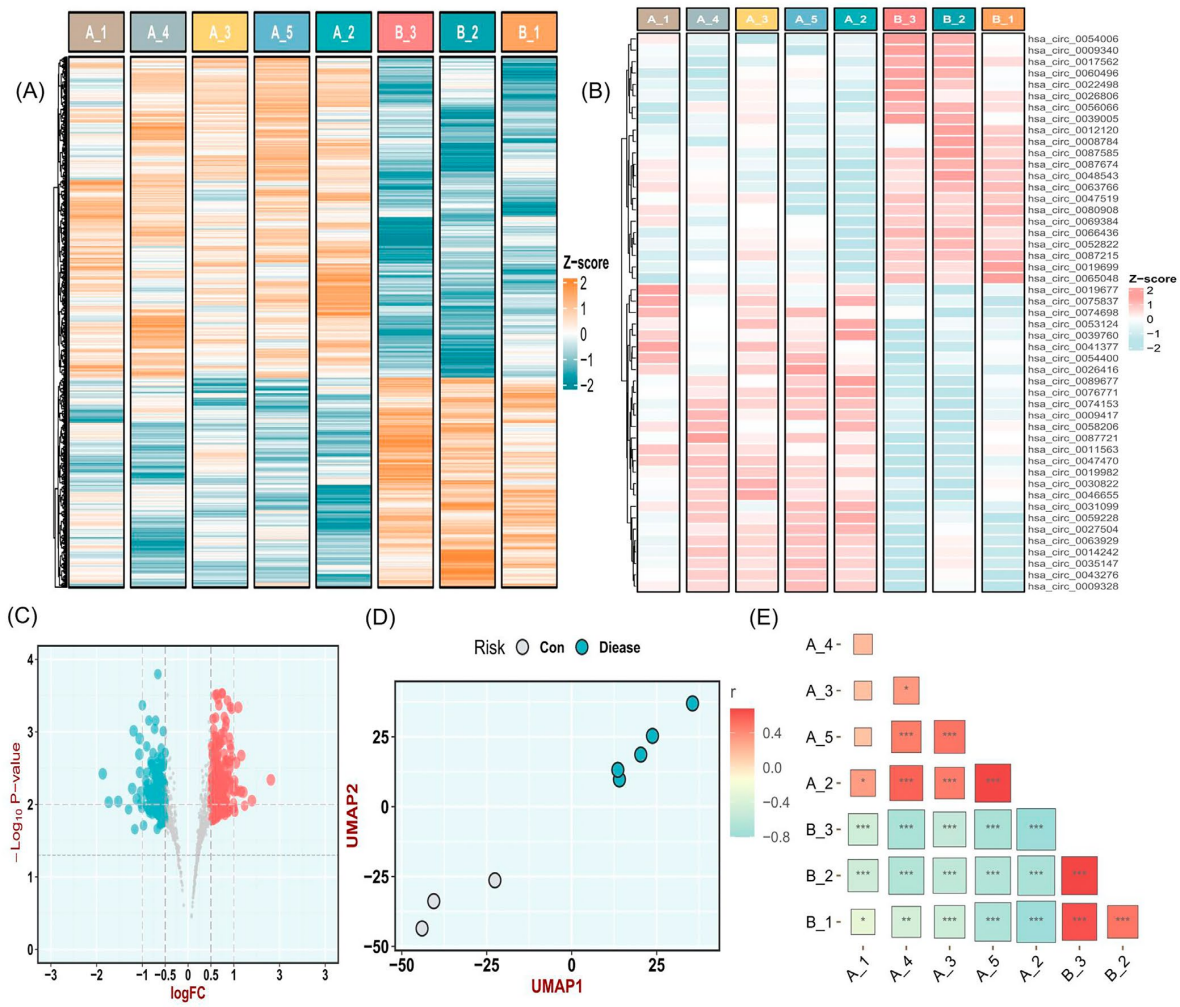
Landscape of exosomal circRNA profile. (A) Heatmap of all expressed exosomal circRNAs between DCM with CHF and controls, where A represented the DCM group and B represented the control group. (B) The heat map shows the 49 circRNA expression profile under the threshold absolute logFC > 0.5 and *p* < 0.05, where A represented the DCM group and B represented the control group. (C) The volcano plot of circRNAs. Red dots represent upregulated circRNAs; grey dots not‐significant circRNAs; and blue dots downregulated circRNAs. (D, E) Pearson correlation analysis plot of normal samples and DCM samples.

A total of 865 circRNAs were detected in the plasma exosome of the DCM with CHF patients and healthy controls. The heatmap demonstrated the landscape of circRNA profile (Figure 2A). A total of 615 circRNAs were significantly differentially expressed between the two groups under a threshold of *p* < 0.05. Among them, 338 circRNAs were upregulated and 277 circRNAs were downregulated. Furthermore, 58 circRNAs were borderline significantly differentially expressed (*p* = 0.05–0.1), including 42 upregulated and 16 downregulated in DCM with HF patients. Based on *p*‐value < 0.05 and |log_2_‐fold change (FC)| > 0.5, we determined 49 DCM‐Exo‐circRNAs for subsequent analysis, including 27 upregulated and 22 downregulated circRNAs (Figure 2B, Table 1). The details of downregulated circRNAs were further demonstrated in the volcano map and bar plot (Figure 2C,D). We further performed the Pearson correlation analysis and UMAP analysis on the circRNA profiles to evaluate the differences and similarities in the circRNA expression between the samples of DCM with CHF patients and healthy control participants (Figure 2E). These results have revealed a significant difference between DCM and healthy individuals on their plasma exosomal circRNA landscapes.

**TABLE 1:** Information of the 49 DCM‐exosome‐circRNAs in DCM with CHF.

	log_2_‐FoldChange	*p*
Upregulation circRNA		
hsa−circ−0074698	1.81	0.02
hsa−circ−0054400	1.40	0.04
hsa−circ−0063929	1.23	0.04
hsa−circ−0053124	1.21	0.03
hsa−circ−0047470	1.18	0.00
hsa−circ−0074153	1.16	0.01
hsa−circ−0075837	1.15	0.03
hsa−circ−0076771	1.10	0.00
hsa−circ−0087721	1.06	0.03
hsa−circ−0011563	1.05	0.01
hsa−circ−0030822	1.02	0.04
hsa−circ−0019677	1.01	0.02
hsa−circ−0058206	1.00	0.04
hsa−circ−0041377	1.00	0.03
hsa−circ−0014242	0.99	0.04
hsa−circ−0046655	0.98	0.02
hsa−circ−0035147	0.97	0.04
hsa−circ−0043276	0.96	0.01
hsa−circ−0009328	0.95	0.00
hsa−circ−0059228	0.94	0.01
hsa−circ−0031099	0.94	0.01
hsa−circ−0089677	0.93	0.03
hsa−circ−0019982	0.92	0.00
hsa−circ−0026416	0.92	0.01
hsa−circ−0027504	0.91	0.02
hsa−circ−0009417	0.90	0.01
hsa−circ−0039760	0.90	0.01
Downregulation‐circRNA		
hsa−circ−0056066	−1.87	0.02
hsa−circ−0026806	−1.74	0.04
hsa−circ−0080908	−1.53	0.04
hsa−circ−0060496	−1.30	0.04
hsa−circ−0066436	−1.22	0.03
hsa−circ−0087585	−1.19	0.01
hsa−circ−0047519	−1.17	0.04
hsa−circ−0063766	−1.12	0.03
hsa−circ−0019699	−1.07	0.01
hsa−circ−0087215	−1.06	0.01
hsa−circ−0009340	−1.05	0.02
hsa−circ−0039005	−1.03	0.03
hsa−circ−0008784	−1.02	0.04
hsa−circ−0022498	−1.01	0.04
hsa−circ−0017562	−1.00	0.00
hsa−circ−0069384	−0.97	0.04
hsa−circ−0012120	−0.93	0.02
hsa−circ−0048543	−0.93	0.02
hsa−circ−0054006	−0.91	0.03
hsa−circ−0065048	−0.90	0.01
hsa−circ−0052822	−0.90	0.04
hsa−circ−0087674	−0.90	0.02

### 3.3 Analysis of Target Genes of DCM‐Exosome‐circRNAs

To predict the potential targets of the 49 identified circRNAs, we utilised two target prediction algorithms, TargetScan and miRanda. The selection criteria were stringent, focusing on the scoring parameters of each software: targets with a TargetScan context score percentile below 80 and those with a miRanda maximum energy threshold of −80 or higher were systematically excluded from consideration. Subsequently, we intersected the refined datasets from both algorithms to determine the definitive target genes for these circRNAs. This meticulous process culminated in the identification of 25 candidate genes, as shown in Table S2. These genes are considered to play pivotal roles in the pathogenic regulatory mechanisms underlying DCM, thus warranting further exploration.

### 3.4 Metascape Functional Enrichment Analysis of DCM‐Exosome‐circRNAs

**FIGURE 3:**
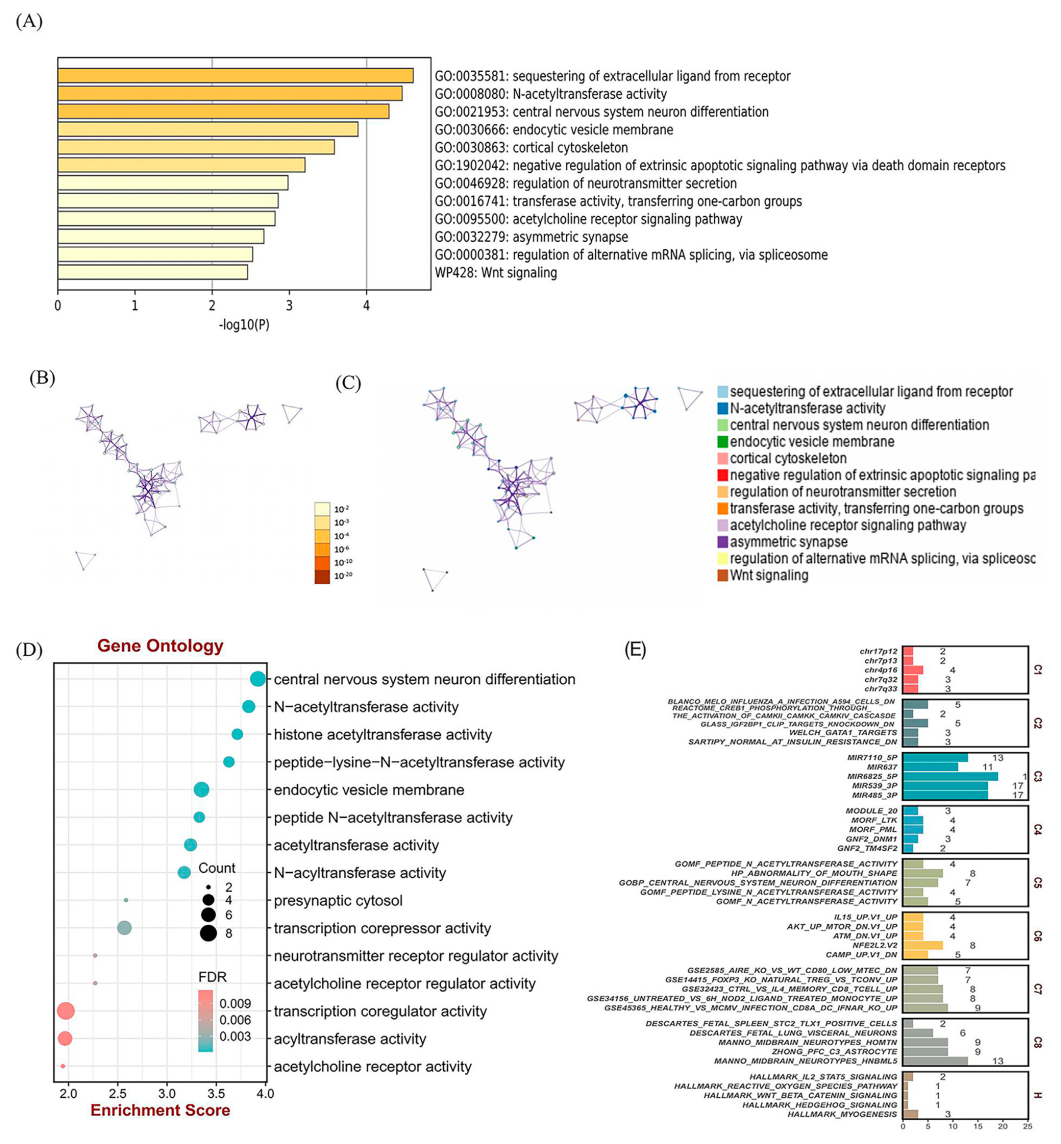
Biological implications of circRNA target genes. (A) Top 12 clusters from Metascape pathway and process enrichment analysis of circRNA target genes, coloured by *p*‐values. (B) Network of enriched terms of circRNA target genes coloured by enriched terms. (C) Network of enriched terms of circRNA target genes coloured by *p*‐value. (D) Bubble plot of GO enrichment analysis of circRNA target genes. (E) Comprehensive enrichment landscape underlying PPI. C1: positional gene sets; C2: curated gene sets; C3: regulatory target gene sets; C4: computational gene sets; C5: ontology gene sets; C7: immunologic signature; C8: cell‐type signature gene sets.

Subsequent to the initial analysis, we conducted a comprehensive Metascape pathway and process enrichment analysis on the predicted targets of DCM‐exosome‐circRNAs. This analysis integrated data from multiple ontology sources, including GO biological processes, GO molecular functions, KEGG pathway, hallmark gene sets, reactome gene sets and canonical pathways. The results highlighted several significantly enriched terms, including sequestering of extracellular ligand from receptor (GO:0035581), N‐acetyltransferase (NAT) activity (GO:0008080) and endocytic vesicle membrane (GO:0030666). The top 12 enriched terms, representing key clusters in this analysis, are displayed in Figure 3A. The process of sequestering extracellular ligands from receptors is crucial for regulating cellular signalling. For instance, in heart failure, the dysregulation of ligand–receptor interactions, such as elevated catecholamine levels binding to β‐adrenergic receptors, exacerbates cardiac dysfunction. The NAT activity is significant in drug metabolism, inflammatory response regulation and oxidative stress, all of which are relevant to cardiovascular diseases [35]. Variations in NAT can affect the metabolism of cardiovascular medications and endogenous compounds, influencing disease progression. Studies have shown that NAT enzymes play a role in the metabolism of polyamines, which are involved in myocardial cell survivability and cardiac remodelling [36]. Our findings are consistent with these studies, indicating that exosome‐derived circRNAs may exert cardioprotective effects through the NAT pathway. The endocytic vesicle membrane pathway is involved in cholesterol regulation, inflammatory responses and endothelial cell function, all critical in cardiovascular diseases. Research has demonstrated its relevance in cardioprotective signal transduction and mitochondrial dysfunction, which are pivotal in heart failure pathology. To elucidate the interrelationships among these top terms, we constructed a functional annotation network, as depicted in Figure 3B,C. To validate the accuracy and reliability of the predicted target genes, we conducted a gene set analysis on DCM‐exosome‐circRNAs using C1‐C8 + hallmark gene sets from the MsigDB database (Table S3).

### 3.5 GO Enrichment Analysis of Target Genes of DCM‐Exosome‐circRNAs

Then, GO software was used for the functional annotation of the 25 predicted target genes of the DCM‐exosome‐derived circRNAs. The GO analysis categorises results into three primary domains: (i) biological process, (ii) cellular component and (iii) molecular function. The analysis revealed that the target genes of DCM‐exosome‐derived circRNAs were significantly enriched in key cellular components, including asymmetric synapse, neuron‐to‐neuron synapse and postsynaptic density (Figure 3D). This suggests that the target genes are involved in critical synaptic functions and neural signalling pathways. The asymmetric synapse component is known to play a role in synaptic plasticity, which has been implicated in the cardiac autonomic regulation [37]. Disruption in synaptic signalling could contribute to the autonomic imbalance observed in DCM patients. Additionally, neuron‐to‐neuron synapse and postsynaptic density are crucial for signal transduction and neuronal communication, processes that can affect cardiac function through neurocardiac interactions. These insights provide a deeper understanding of how circRNAs might influence the neuroregulatory mechanisms in DCM, highlighting potential targets for therapeutic intervention. This enrichment in specific cellular components suggested a potential mechanistic link to the pathogenesis and progression of DCM with CHF. Figure 3E illustrates that, in alignment with our data sources, these targets were significantly enriched in the C3 regulatory target gene sets. This finding suggests that the predicted target genes are closely associated with the plasma exosomal circRNAs in DCM with CHF. This network offered insights into the potential interconnected pathways and processes influenced by the DCM‐exosome‐derived circRNAs, thereby deepening our understanding of their roles in the molecular mechanisms underlying DCM.

### 3.6 Protein–Protein Interaction Network Analysis of DCM‐Exosome‐circRNAs

**FIGURE 4:**
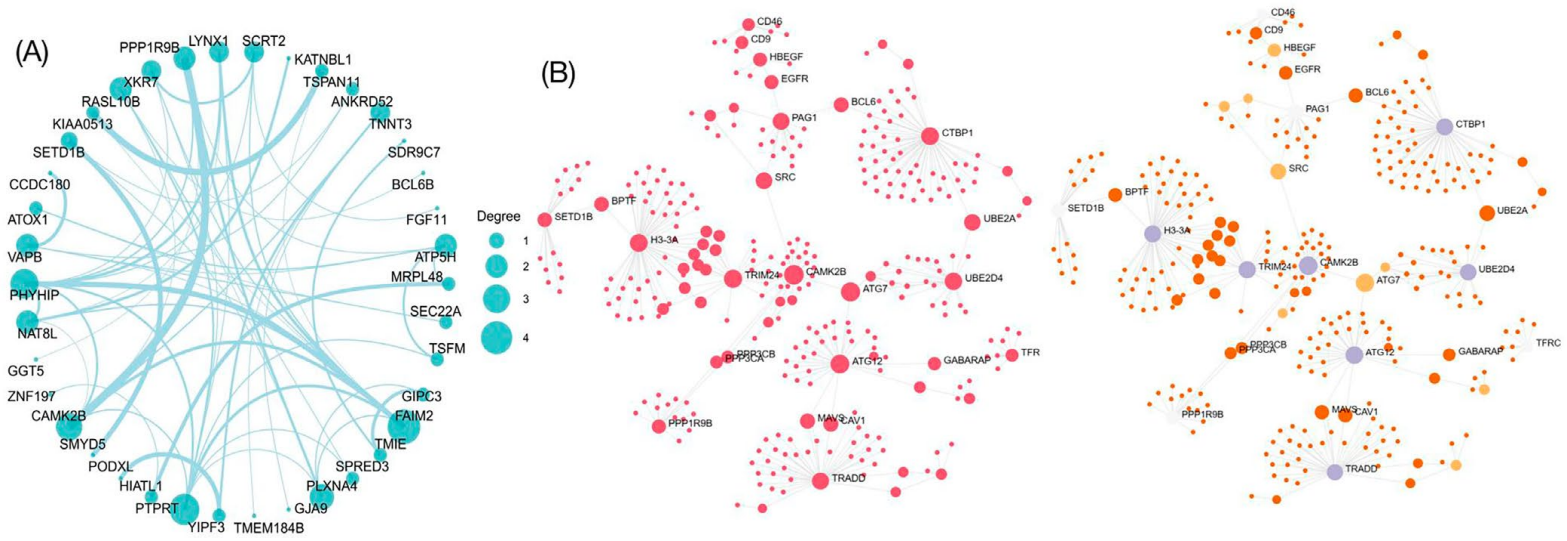
(A) Gene correlation network analysis to identify hub genes in DCM accompanied with CHF. Every node represents one gene, and each edge represents the interaction between nodes. Protein–protein interaction network of target genes of circRNAs and 38 hub genes (degree > 5) were annotated. Coloured and sized by degree (number of connections). (B) Protein–protein interaction network of target genes of circRNAs in InnateDB.

We imported the target genes of DCM‐exosome‐circRNAs, identified through TargetScan and miRanda, into the STRING database for PPI network analysis. After eliminating isolated target genes lacking interactions, a total of 38 target genes were incorporated into the PPI network, with a confidence score cut‐off value of 900. The gene interactions were visualised using Cytoscape software (Figure 4A). Employing the CytoHubba plugin, we determined 25 nodes with a degree greater than 5 as hub genes within the PPI network, which are listed in Table 2. We also used InnateDB (https://www.ncbi.nlm.nih.gov/pmc/articles/PMC3531080/) Software validation of gene interactions (Figure 4B). These hub genes are pivotal to numerous biological processes, including signal transduction, metabolic pathways and cellular structural integrity. Notably, the identified hub genes encompass those implicated in cardiac muscle contraction, stress response and apoptotic mechanisms, which are essential in heart failure and cardiomyopathy. Previous research has demonstrated that genes involved in calcium signalling, including those encoding calcium channels and transporters, are integral to cardiac function [38]. The disruption of calcium homeostasis is one of the hallmarks of heart failure. Our findings indicate that circRNAs may modulate these crucial pathways, providing novel insights into their roles in DCM pathophysiology and identifying potential therapeutic targets.

**TABLE 2:** Topology parameters of hub genes (degree > 5) in the PPI network.

Hub Gene	Degree	Hub Gene	Degree
CAMK2B	8	BCL6B	1
FAIM2	8	FGF11	1
PLXNA4	8	GJA9	1
PTPRT	8	KATNBL1	1
NAT8L	7	MRPL48	1
PHYHIP	7	PODXL	1
RASL10B	7	SDR9C7	1
PPP1R9B	6	TMEM184B	1
VAPB	5	TMIE	1
XKR7	5	TNNT3	1
ATP5H	3	YIPF3	1
LYNX1	3	ZNF197	1
SCRT2	3		

### 3.7 Validation of Hub circRNAs Performance in an External Cohort

**FIGURE 5:**
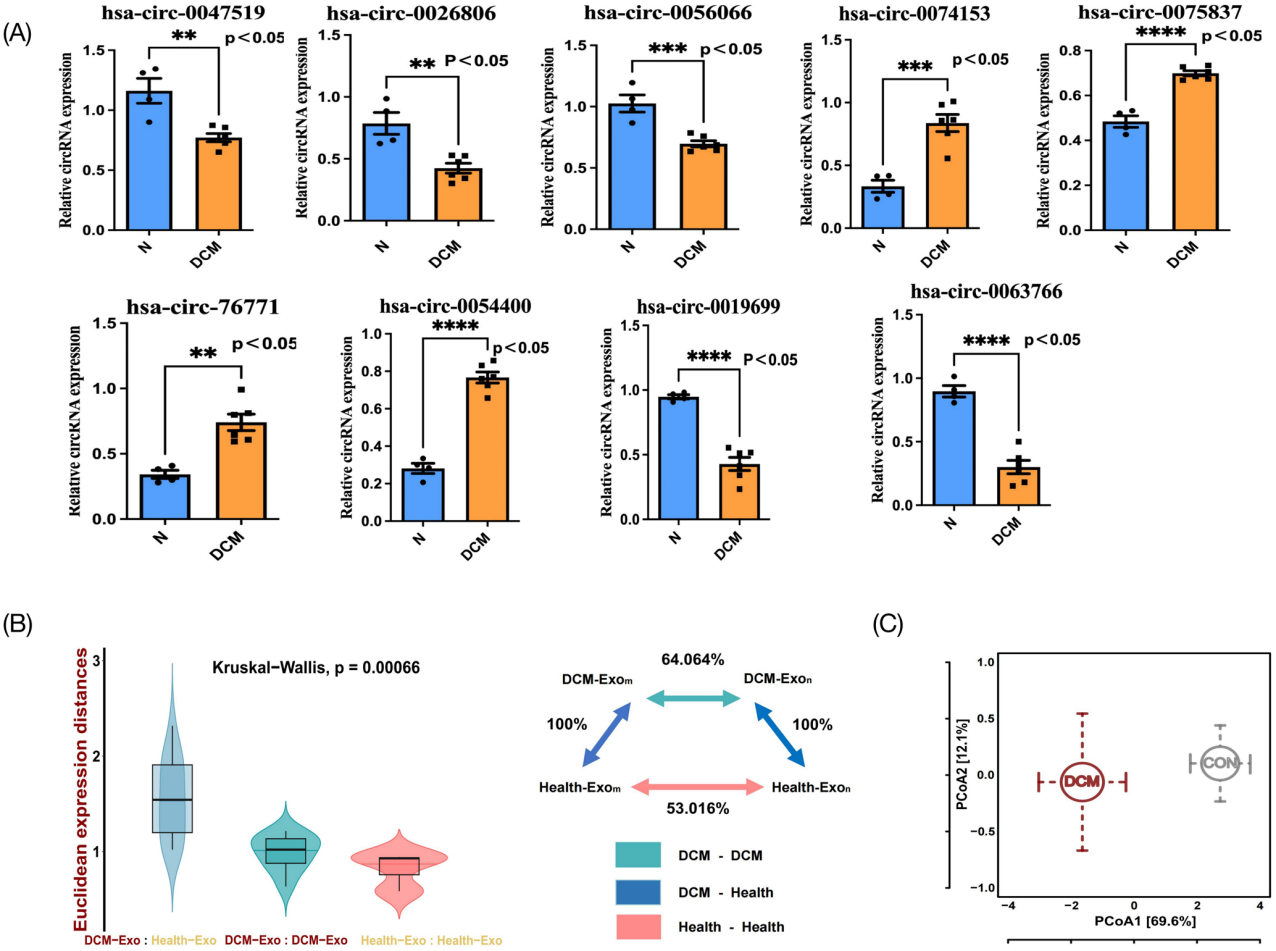
(A) The distribution of nine hub circRNAs expression level between DCM and healthy control groups in the external cohort based on qRT‐PCR. *p < 0.05 (Wilcoxon test); ns represents no significance. (B) Global differences in nine hub circRNA expression between DCM and healthy control groups in the external cohort. The Euclidean expression distances were calculated between DCM and controls (blue), different samples of DCM (green) and different samples of controls (pink). The inset summarises the average distances between pairs of samples as a percentage of the average distance between DCM and controls. (C) Principle coordinate analysis of the Bray–Curtis dissimilarities obtained for the nine hub circRNA expression profiles in the external (open circles) cohorts. The circles and error bars indicated the mean and standard errors of the mean.

To validate our findings, we identified the top nine significantly dysregulated circRNAs in DCM patients compared with healthy individuals through in silico analysis as hub circRNAs. Specifically, these circRNAs were hsa‐circ‐0047519, hsa‐circ‐0063766, hsa‐circ‐76,771, hsa‐circ‐0054400, hsa‐circ‐0019699, hsa‐circ‐0074153, hsa‐circ‐0075837, hsa‐circ‐0056066 and hsa‐circ‐0026806. The expressions of these hub circRNAs were subsequently validated in an external cohort comprising exosomes from six DCM patients and four healthy individuals, using quantitative RT‐PCR. Our results confirmed significant dysregulation of these hub circRNAs in DCM samples compared to controls (Figure 5A). We utilised the Euclidean distance as a measure of divergence between expression profiles to investigate global shifts in the hub circRNA expression between DCM patients and healthy controls within an external cohort. This metric revealed consistent relative differences in expression divergence. Specifically, the expression distance between DCM patients and healthy controls, as well as among DCM samples, was significantly greater than the distance observed within healthy controls (Figure 5B). PCoA of hub circRNA profiles in our internal in silico cohort demonstrated distinct separation between DCM and healthy controls. These findings were corroborated by our external independent cohort, which also showed significant differences in hub circRNA profiles between the two groups (Figure 5C).

## 4 Discussion

In this study, we have delineated specific circRNA characteristics in plasma exosomes of patients suffering from DCM with CHF. Through ceRNA sequencing in DCM patients with CHF, 49 differentially expressed circRNAs were identified, including 27 upregulated and 22 downregulated circRNAs, along with 38 targeted interaction sites. Employing GO and KEGG enrichment analyses, we predicted the regulatory functions of these circRNAs in DCM associated with CHF. These analysis results highlighted key pathways, particularly enriched in the sequestering of extracellular ligand from receptor, NAT activity and endocytic vesicle membrane. This research sheds light on the molecular mechanisms in DCM with CHF and suggests potential therapeutic targets.

Sequestering of extracellular ligand from receptor is a pivotal biological mechanism by which certain molecules, typically proteins, capture or sequester specific ligands extracellularly, thus preventing their binding to target receptors [39]. This process is crucial in regulating cellular signal transduction and is involved in various diseases, including cardiovascular disease. In heart failure, a clinical syndrome marked by diminished cardiac function, the interaction of extracellular ligands with receptors plays a crucial role in pathogenesis. For instance, prolonged elevated levels of catecholamines, which bind to β‐adrenergic receptors, can exacerbate heart failure [40]. Consequently, strategies like β‐adrenergic receptor blockers, which reduce cardiac workload by modulating ligand–receptor interactions, have become standard treatments for heart failure. Furthermore, our study explored several mechanisms of extracellular ligand sequestration. These include using antagonists or inhibitors like β‐adrenergic receptor blockers and neutralising antibodies that bind and neutralise specific ligands and soluble receptors that bind to ligands without activating them. The high affinity of BA for TGF‐β1, as reported by Lan et al., exemplifies an efficient sequestration mechanism [41]. Similarly, Sugden and colleagues highlighted the role of ErbB receptor/ligand in myocardial function, noting their influence on cardiac myocyte survivability and hypertrophy [42]. Camila's team pointed out the significance of the P2X7 receptor in cardiovascular diseases, emphasising its potential as a pathological marker and therapeutic target [43]. These insights underscore the importance of understanding extracellular ligand sequestration mechanisms for developing effective cardiovascular disease treatments. Considering the multifactorial nature of DCM with heart failure, a combination of extracellular ligand sequestration strategies with lifestyle modifications, pharmacotherapy and surgical interventions is essential for optimal treatment. Our findings suggest that circRNAs involved in the extracellular ligand–receptor sequestration pathway could offer cardioprotective effects in DCM patients with CHF.

The NAT activity, which is crucial in drug metabolism, inflammatory response regulation and oxidative stress reactions, plays a significant role in cardiovascular diseases [44]. Variations in NAT affect the metabolism of various cardiovascular medications and inflammatory mediators and also influence the metabolism of endogenous and exogenous compounds impacting cardiovascular health [45]. For instance, the metabolism of environmental toxins via NAT pathways can have cardiovascular implications [46]. Furthermore, NAT enzymes partake in the metabolism of hormones that indirectly influence cardiovascular health [47]. Zhao et al.'s research on Spermidine/spermine N(1)‐acetyltransferase (SSAT) in myocardial ischemia in rats revealed its involvement in polyamine metabolism regulation and its impact on myocardial cell survivability [48]. Similarly, Shi et al.'s discovery of the upregulation of NAT 10 (NAT10) in cardiac hypertrophy highlights NAT10's role in enhancing mRNA stability and translation efficiency of genes implicated in cardiac remodelling [44]. Our study aligns with these findings, indicating the protective effect of circRNAs in exosomes on myocardial tissue through the NAT pathway in DCM with CHF.

The endocytic vesicle membrane pathway, a cellular process involving the formation and utilisation of endosomes, is also implicated in the onset and progression of cardiovascular diseases [49]. It plays a role in cholesterol regulation, inflammatory responses, endothelial cell function, oxidative stress response and cell death regulation [50]. Studies by Sauri Hernández‐Reséndiz et al. [51], Cui et al. [52] and Yang's team [53] have highlighted the pathway's relevance in cardiovascular diseases, particularly in cardioprotective signal transduction and mitochondrial dysfunction. Our study corroborates these findings, revealing the critical role of circRNAs in exosomes via the endocytic vesicle membrane pathway in DCM with HF.

Furthermore, we identified nine dysregulated exosomal circRNAs associated with DCM and CHF. These circRNAs, validated in DCM patient plasma samples, may play significant roles in inflammation, oxidative stress, cellular vesicle trafficking and NAT activity [6, 54]. This identification laid the foundation for future research to elucidate the pathophysiological mechanisms of these circRNAs in DCM, potentially leading to new therapeutic targets and improved patient outcomes.

However, our study has limitations. The small sample size and the specific patient population limit the generalisability of our findings. The direct cardiac origin of the circulating circRNAs and the mechanisms by which the differentially expressed exosomal circRNAs contribute to DCM pathophysiology still require further investigation.

## 5 Conclusion

This study marks a significant advancement in understanding the pathophysiological mechanisms of DCM with CHF, highlighting the crucial role of circRNAs in plasma exosomes. Our investigation, employing next‐generation sequencing technology, has revealed distinct circRNA expression patterns in DCM patients, identifying 49 uniquely expressed circRNAs associated with critical biological pathways such as extracellular ligand sequestration, NAT activity and endocytic vesicle membrane composition. These findings not only deepen our understanding of DCM's molecular underpinnings but also provide the novel diagnostic and therapeutic method. Moreover, the elucidation of their roles in sequestering extracellular ligands, modulating NAT activity and influencing endocytic vesicle membrane processes offers a new perspective on the disease's pathogenesis, providing a basis for targeted therapeutic interventions. In conclusion, the insights gained from this research contribute substantially to the translational medicine field, offering promising ways for enhancing the clinical management of DCM. By integrating these molecular findings with clinical practice, we move a step closer to personalised medicine, where targeted therapies and precise diagnostics can significantly improve patient outcomes in DCM and potentially other forms of heart failure.

## Author Contributions

**Shuai Xu, Ge Zhang, Xin Tan and Yiyao Zeng** indicates equal contribution. **Shuai Xu:** conceptualization (equal), data curation (equal), formal analysis (equal), funding acquisition (equal), investigation (equal), methodology (equal), project administration (equal), resources (equal), software (equal), supervision (equal), validation (equal), visualization (equal), writing – original draft (equal), writing – review and editing (equal). **Ge Zhang:** conceptualization (equal), data curation (equal), formal analysis (equal), funding acquisition (equal), investigation (equal), methodology (equal), project administration (equal), resources (equal), software (equal), supervision (equal), validation (equal), visualization (equal), writing – original draft (equal), writing – review and editing (equal). **Xin Tan:** conceptualization (equal), data curation (equal), formal analysis (equal), funding acquisition (equal), investigation (equal), methodology (equal), project administration (equal), resources (equal), software (equal), supervision (equal), validation (equal), visualization (equal), writing – original draft (equal), writing – review and editing (equal). **Yiyao Zeng:** conceptualization (equal), data curation (equal), formal analysis (equal), funding acquisition (equal), investigation (equal), methodology (equal), project administration (equal), resources (equal), software (equal), supervision (equal), validation (equal), visualization (equal), writing – original draft (equal), writing – review and editing (equal). **Huimin Fan:** validation (equal), visualization (equal), writing – original draft (equal), writing – review and editing (equal). **Jiamin Gao:** validation (equal), visualization (equal), writing – original draft (equal), writing – review and editing (equal). **Zhen Qin:** validation (equal), visualization (equal), writing – original draft (equal), writing – review and editing (equal). **Fengyi Yu:** validation (equal), visualization (equal), writing – original draft (equal), writing – review and editing (equal). **Bin Ma:** validation (equal), visualization (equal), writing – original draft (equal), writing – review and editing (equal). **Ting Zhang:** validation (equal), visualization (equal), writing – original draft (equal), writing – review and editing (equal). **Hezi Jiang:** validation (equal), visualization (equal), writing – original draft (equal), writing – review and editing (equal). **Xian Li:** validation (equal), visualization (equal), writing – original draft (equal), writing – review and editing (equal). **Xiangyu Wang:** validation (equal), visualization (equal), writing – original draft (equal), writing – review and editing (equal). **Jili Fan:** validation (equal), visualization (equal), writing – original draft (equal), writing – review and editing (equal). **Xiaohong Bo:** validation (equal), visualization (equal), writing – original draft (equal), writing – review and editing (equal). **Yafeng Zhou:** conceptualization (lead), data curation (lead), formal analysis (lead), funding acquisition (lead), investigation (lead), methodology (lead), project administration (lead), resources (lead), software (lead), supervision (lead), validation (lead), visualization (lead), writing – original draft (lead), writing – review and editing (lead). **Junnan Tang:** conceptualization (lead), data curation (lead), formal analysis (lead), funding acquisition (lead), investigation (lead), methodology (lead), project administration (lead), resources (lead), software (lead), supervision (lead), validation (lead), visualization (lead), writing – original draft (lead), writing – review and editing (lead).

## Conflicts of Interest

The authors declare no conflicts of interest.

## Supporting information


**Table S1.** Clinical features of all participants.

**Table S2.** Predicted target genes of the 49 circRNAs, with 25 candidate genes identified using TargetScan and miRanda algorithms.

**Table S3.** Gene set analysis of DCM‐exosome‐circRNAs using C1‐C8 and hallmark gene sets from the MsigDB database.

## Data Availability

The datasets used and/or analysed during the current study are available from the corresponding author on reasonable request All data are available in a public, open access repository. R and other custom scripts for analysing data are available upon reasonable request.
