# Supplementary material for: Differential Expression Spectrum of circRNA in Plasma Exosomes in Dilated Cardiomyopathy With Heart Failure
Source: J Cell Mol Med. 2024 Dec 24;28(24):e70258. doi: 10.1111/jcmm.70258 (PMC11668728; doi:10.1111/jcmm.70258)
Supplement: Supplementary file 1 — Table S1. Clinical features of all participants. [file JCMM-28-e70258-s001.docx]

Table S1. Clinical Features of all participants

| Characteristics | Healthy Group | DCM Group | *p* |
| --- | --- | --- | --- |
| Sample sizes | N=5 | N=3 |  |
| Age (years) | 34.00(34.00-35.00) | 34.00(31.00-42.00) | 0.647 |
| Gendern |  |  |  |
| Male, n(%) | 3(60.00) | 2(66.67) | 0.237 |
| Laboratory parameters |  |  |  |
| WBC, 10^9^/L | 7.28 (6.64-8.11) | 7.15 (6.45-7.35) | 0.655 |
| Neut, 10^9^/L | 5.44 (4.65-5.72) | 3.29 (3.24-3.81) | 0.053 |
| Hb, g/L | 129.60 (113.31-150.07) | 135.53 (123.39-145.53) | 0.654 |
| PLT, 10^9^/L | 275.92 (209.72-281.41) | 256.49 (217.31-261.66) | 0.297 |
| Albumin(g/L) | 38.91 (38.32-40.14) | 42.74 (41.95-44.97) | 0.180 |
| TC (mmol/L) | 4.16 (4.05-4.95) | 3.49 (3.33-3.77) | 0.101 |
| TG (mmol/L) | 1.23(0.88-1.52) | 0.82(0.60-1.427) | 0.162 |
| LDL-C(mmol/L) | 0.98 (0.98-1.16) | 3.88 (3.54-4.31) | 0.024 |
| Urea(mmol/L) | 4.08 (3.41-4.49) | 4.36 (3.96-5.75) | 0.456 |
| CRP(mg/L) | 0.28 (0.24-0.36) | 0.98 (0.67-1.31) | 0.072 |
| NT-proBNP(pg/ml) | 74.00 (67.00-88.00) | 1493.05 (1217.36-1538.39) | 0.025 |
| LVEF (%) | 73.00 (70.00-75.00) | 45.00 (36.00-50.00) | 0.025 |
| Hypertension,n(%) | 4(80.00) | 1(33.33) | 0.217 |

Abbreviations:DCM, Dilated Cardiomyopathy; WBC, white blood cell; Neut,neutrophils;Hb,hemoglobin; PLT, Platelet; TC, total cholesterol; TG,triglyceride; LDL-C, low-density lipoprotein cholesterol; CRP, C-reactive proteine; NT-proBNP, N-terminal pro-B-type natriuretic peptide; LVEF, left ventricular ejection fractions.
